# Targeting FGFR in non-small cell lung cancer: implications from the landscape of clinically actionable aberrations of FGFR kinases

**DOI:** 10.20892/j.issn.2095-3941.2020.0120

**Published:** 2021-06-15

**Authors:** Zhen Zhou, Zichuan Liu, Qiuxiang Ou, Xue Wu, Xiaonan Wang, Yang Shao, Hongyan Liu, Yu Yang

**Affiliations:** 1 Shanghai Lung Cancer Center, Shanghai Chest Hospital, Shanghai Jiao Tong University, Shanghai 200030, China; 2 Section No. 2 Internal Medicine, Cancer Center of Guangzhou Medical University, Guangzhou 511436, China; 3 Translational Medicine Research Institute, Geneseeq Technology Inc., Toronto M5G1L7, Canada; 4 Nanjing Geneseeq Technology Inc., Nanjing 211500, China; 5 School of Public Health, Nanjing Medical University, Nanjing 211166, China; 6 Department of Respiratory Medicine, The Second Hospital of Anhui Medical University, Hefei 230031, China; 7 Department of Oncology, The Second Affiliated Hospital of Harbin Medical University, Harbin Medical University, Harbin 150086, China

**Keywords:** *FGFR*, oncogenic mutation, fusion, gene amplification, targeted therapy

## Abstract

**Objective:**

Dysfunction in fibroblast growth factor receptor (FGFR) signaling has been reported in diverse cancer types, including non-small cell lung cancer (NSCLC). The frequency of *FGFR* aberrations in Chinese NSCLC patients is therefore of great clinical significance.

**Methods:**

A total of 10,966 NSCLC patients whose tumor specimen and/or circulating cell-free DNA (cfDNA) underwent hybridization capture-based next-generation sequencing were reviewed. Patients’ clinical characteristics and treatment histories were also evaluated.

**Results:**

*FGFR* aberrations, including mutations, fusions, and gene amplifications, were detected in 1.9% (210/10,966) of the population. *FGFR* abnormalities were more frequently observed in lung squamous cell carcinomas (6.8%, 65/954) than lung adenocarcinomas (1.3%, 128/9,596). *FGFR* oncogenic mutations were identified in 19 patients (∼0.17%), of which, 68% were male lung squamous cell carcinoma patients. Eleven out of the 19 patients (58%) had concurrent altered PI3K signaling, thus highlighting a potential combination therapeutic strategy of dual-targeting FGFR and PI3K signaling in such patients. Furthermore, *FGFR* fusions retaining the intact kinase domain were identified in 12 patients (0.11%), including 9 *FGFR3-TACC3*, 1 *FGFR2-INA*, 1 novel *FGFR4-RAPGEFL1*, and 1 novel fusion between the *FGFR1* and *SLC20A2* 5′-untranslated regions, which may have caused *FGFR1* overexpressions. Concomitant *EGFR* mutations or amplifications were observed in 6 patients, and 4 patients received anti-EGFR inhibitors, in whom *FGFR* fusions may have mediated resistance to anti-EGFR therapies. *FGFR* amplification was detected in 24 patients, with the majority being *FGFR1* amplifications. Importantly, *FGFR* oncogenic mutations, fusions, and gene amplifications were almost always mutually exclusive events.

**Conclusions:**

We report the prevalence of *FGFR* anomalies in a large NSCLC population, including mutations, gene amplifications, and novel *FGFR* fusions.

## Introduction

The fibroblast growth factor/fibroblast growth factor receptor (FGF/FGFR) signaling pathway plays important roles in a variety of biological processes, including development, differentiation, cell proliferation, migration, angiogenesis, and carcinogenesis *via* several intracellular pathways, including the Ras/Raf/MEK and the phosphatidylinositol 3-kinase (PI3K)-AKT pathways^1^. The FGF family contains 22 members, which are usually divided into 7 subfamilies according to their sequence similarities, biochemical functions, and evolutionary relationships^2^. All 4 FGFRs, including FGFR1, FGFR2, FGFR3, and FGFR4 are structurally homologous to vascular endothelial growth factor receptors (VEGFRs), platelet-derived growth factor receptor (PDGFR), and other tyrosine kinase receptors^3^, and represent therapeutic targets of great potential.

Previous studies have shown that *FGFR2/3* gene alterations, including *FGFR3* activating mutations that affect either the extracellular (R248C and S249C) or transmembrane (G370C, S371C, Y373C, and G380R) domains of the protein, and gene fusions such as *FGFR3-TACC3*, are common in patients with urothelial carcinoma and cause constitutively activated FGF signaling, resulting in carcinogenesis^4^. Multiple FGFR inhibitors^5^, including erdafitinib^6,7^ have shown antitumor activities in preclinical models and in early phase clinical trials involving patients with *FGFR* alterations. A recent study by Loriot et al.^8^ reported that the use of erdafitinib was associated with an objective tumor response in 40% of previously treated patients who had locally advanced and unresectable or metastatic *FGFR* alteration-positive urothelial carcinomas. Such findings were superior to prior observations of an objective response rate of approximately 10% using second-line, single agent chemotherapy in an advanced urothelial carcinoma population^9–11^.

Activation of FGF signaling has also been described in lung cancer, including non-small cell lung cancer (NSCLC). As previously described, the incidence of *FGFR* alterations, particularly *FGFR1* amplification, was higher in squamous cell carcinoma (SCC) of the lung than in adenocarcinoma^12^. Moreover,* FGFR2* mutations were also reported in NSCLC patients, including the extracellular domain mutations, W290C and S320C, and the kinase domain mutation, K660E/N^13^. In this study, we investigated the landscape of *FGFR* aberrations in a large Chinese NSCLC population by comprehensive genomic profiling using next-generation sequencing (NGS), to identify potential therapeutic options for *FGFR*-mutated NSCLC patients.

## Materials and methods

### Patients

A total of 15,150 consecutive clinical lung cancer patients were analyzed using comprehensive genomic profiling targeting 400+ cancer-relevant genes, including all the exons of *FGFR* genes (*FGFR*1-4), as well as flanking intronic regions, and other introns selected by a Clinical Laboratory Improvement Amendments-certified, and College of American Pathologists-accredited laboratory (Nanjing Geneseeq Technology, Jiangsu, China), as previously described^14^. We identified patients with *FGFR* alterations using a natural language search tool in the laboratory information management system database. Relevant demographic and clinical data were extracted from the database, including age, gender, date of diagnosis, histology, pathological stage, and evaluation of treatment response based on reports by clinical investigators.

For tumor tissue samples, the pathological diagnosis and tumor content of each case was confirmed by pathologists. Peripheral blood (8–10 mL) was collected in EDTA-coated tubes (BD Biosciences, San Jose, CA, USA) and centrifuged at 1,800 × *g* for 10 min within 2 h of collection to isolate the plasma for circulating tumor DNA (ctDNA) extraction, and white blood cells for genomic DNA extraction as the germline control.

### DNA extraction and targeted enrichment

The ctDNA from plasma was purified using a Circulating Nucleic Acid Kit (Qiagen, Hilden, Germany) following the manufacturer’s protocol. Genomic DNA from white blood cells was extracted using the DNeasy Blood and Tissue Kit (Qiagen), while genomic DNA from formalin-fixed paraffin-embedded (FFPE) samples was purified using the QIAamp DNA FFPE Tissue Kit (Qiagen). All DNA was quantified using the dsDNA HS Assay Kit using a Qubit Fluorometer (Life Technologies, Carlsbad, CA, USA). Sequencing libraries were prepared using the KAPA Hyper Prep Kit (Roche, Basel, Switzerland), as described previously^14^. Indexed DNA libraries were pooled for probe-based hybridization capture of the targeted gene regions covering over 400 cancer-related genes for all solid tumors; all of which contained all exons of *FGFR* genes and selected introns for the detection of *FGFR* fusions.

### Sequencing data processing

Sequencing was performed using the Illumina HiSeq4000 platform (Illumina, San Diego, CA, USA), followed by data analysis as previously described^15^. In brief, sequencing data were analyzed by Trimmomatic^16^ to remove low quality (quality < 15) or n bases, and were then mapped to the human reference genome, hg19, using the Burrows-Wheeler Aligner (https://github.com/lh3/bwa/tree/master/bwakit). PCR duplicates were removed by Picard (https://broadinstitute.github.io/picard/). The Genome Analysis Toolkit (GATK) (https://software.broadinstitute.org/gatk/) was used to perform local realignments around indels and for base quality reassurance. Single nucleotide polymorphisms (SNPs) and indels were analyzed by VarScan2^17^ and HaplotypeCaller/UnifiedGenotyper in GATK, with the mutant allele frequency cutoff at 0.5% for tissue samples, 0.1% for cfDNA samples, and a minimum of three unique mutant reads. Common SNPs were excluded if they were present in > 1% population frequency in the 1,000 Genomes Project or the Exome Aggregation Consortium (ExAC) 65,000 exome database. The resulting mutation list was further filtered using an in-house list of recurrent artifacts based on a normal pool of whole blood samples. Gene fusions were identified by FACTERA^18^.

### Ethical approval

The study was approved by the Ethics Committee of Guangdong General Hospital, China (Approval No. GDREC2016262H). Shanghai Chest Hospital served as one of the hospitals participating in the research project. The study was conducted in accordance with the tenets of the Declaration of Helsinki, and written informed consent was collected from each patient prior to sample collection.

## Results

### The incidence of *FGFR* aberrations in NSCLC patients

**Figure 1:**
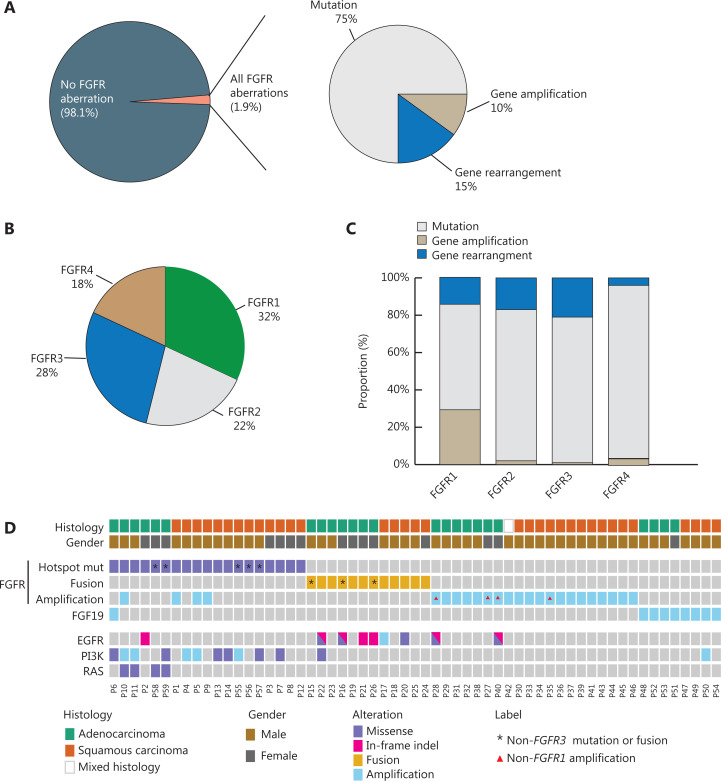
Distribution of *FGFR* aberrations in a large population of Chinese patients with non-small cell lung cancer. (A) The frequency of *FGFR* aberrations among all cases and (B) the relative proportion of *FGFR* aberrations of *FGFR* genes among all cases, with the breakdown of *FGFR* alterations (C). (D) Co-mutation plot showing patients who carried *FGFR* oncogenic mutations, fusions, and gene amplifications, as well as concomitant aberrations of genes, including *EGFR, RAS,* and components of the PI3K pathway. An additional 9 patients with *FGF19* amplifications were also plotted. The asterisk indicates mutations or fusions in *FGFRs* other than *FGFR3*. The triangle indicates non-*FGFR1* amplifications.

From December 2016 to February 2019, a total of 15,150 individual clinical lung cancers were successfully evaluated by comprehensive genomic profiling using hybrid capture-based NGS. This work was based on the validated dataset for a total of 10,966 patients in our database system. Lung cancer tumor samples and liquid biopsies, if applicable, were compared to matched normal whole blood controls. A total of 87% of NSCLC samples examined were lung adenocarcinomas [lung adenocarcinoma (LUAC), *n* = 9,596], 9% were lung squamous cell carcinoma (LUSC, *n* = 954), and the remainder (4%) were of either mixed adenocarcinomas and squamous cell carcinomas or were missing sub-histological information in the database. Approximately 40% of the entire study population had only liquid biopsy specimens for genetic testing. A total of 210 patients (1.9%, 210/10,966) were identified with somatic aberrations of *FGFRs* (*FGFR1–4*), including mutations, gene rearrangements, and gene amplifications (**Figure 1A**). Fifty-one patients (roughly 24%) had liquid biopsy samples including only plasma and pleural effusion samples. The median age of the cohort was 62 years of age (range: 34–84 years of age). Approximately 72% (152/210) of the patients were male. Approximately 61% of *FGFR*-positive patients were LUAC (*n* = 128), 31% were LUSC (*n* = 65), and the remaining 7 cases were of either mixed or unknown histology. Thus, *FGFR* alterations were more frequent in LUSC patients (6.8%, 65/954) than in LUAC patients (1.3%, 128/9,596). The majority of the *FGFR* aberrations were gene mutations (75%) with gene amplification and gene rearrangements being observed in similar frequencies (10% and 15%, respectively) (**Figure 1A**). *FGFR1* alterations were slightly more abundant than alterations in *FGFR2-4* (**Figure 1B**). Notably, we observed more amplification events in *FGFR1s* than in other *FGFRs*, and over 90% of *FGFR4* alterations were mutations (**Figure 1C**).

### Enrichment of the activated PI3K pathway in the *FGFR* mutant cohort

We identified a total of 187 patients with somatic point mutations and indels in* FGFR*s. The most frequent amino acid replacements across all FGFRs were FGFR3 S249C and R248C (**Supplementary Figure S1**). In particular, 19 patients representing ∼0.17% (19/10,966) of the NSCLC population were identified with *FGFR1-4* oncogenic or likely oncogenic mutations according to the OncoKB database^19^ (**Figure 1D**, **Table 1,** and **Supplementary Table S1**). The majority of these patients (68%, 13/19) had lung squamous cell carcinoma, and two-thirds were male. Intriguingly, more than half of the 19 patients (58%, 11/19) had co-occurring *PIK3CA* aberrations, including *PIK3CA* E545K (*n* = 3), E453K (*n* = 1), H1049R (*n* = 1), A1035T (*n* = 1), *PIK3CA* amplifications (*n* = 4), and *PIK3R2* G373R (*n* = 1) mutations. One patient had a concurrent activating *EGFR* ex19del, 4 patients had *KRAS* G12D/V or Q61L mutations, and the remaining 6 patients had no other known driver mutations (**Table 1**). A majority of the 19 patients with *FGFR1-4* oncogenic mutations (68%, 13/19) were systemic treatment-naïve, with the exception that 1 patient progressed on multiple lines of EGFR tyrosine kinase inhibitors 9TKIs0, including gefitinib, osimertinib, and afatinib, and 5 patients either received multiple lines of chemotherapy or chemotherapy in combination with radiotherapy or VEGFR antibody therapy (**Table 1**). Notably, the patient (P2) who received multiple EGFR TKIs likely acquired* FGFR3* R248C and/or G380R to overcome the anti-tumor activity of TKIs, including osimertinib and afatinib, although pretreatment samples were unfortunately not available (**Table 1**).

**Table 1:** The demographical and clinicopathological characteristics of patients who had* FGFR* oncogenic mutations

ID	Subtype	Gender	Age	Stage	Treatment history [TKI (PFS)]	Gene	AAChange	AF	Concurrent alteration	AF_concurrent alt	CNV	Sample type
P1	LUSC	M	56	NA	Chemo, radiotherapy	FGFR3	c.746C>G(p.S249C)	2.51%	−	−	−	Plasma
P2	LUAC	F	52	IV	Gefitinib (21 m), chemo plus VEGFR ab (4 m), osimertinib (5 m), afatinib (5 m)	FGFR3	c.742C>T(p.R248C), c.1138G>A(p.G380R)	5.52%, 4.94%	EGFR c.2240_2257delTAAGAGAAGCAACATCTC (p.L747_P753delinsS), EGFR T790M	4.3%, 1.3%	−	Plasma (post gefitinib)
P3	LUSC	F	66	NA	Surgery	FGFR3	c.746C>G(p.S249C)	31.64%	−	−	−	FFPE
P4	LUSC	M	67	NA	Chemo	FGFR3	c.746C>G(p.S249C)	33.33%	PTEN p.K147Rfs*6, PIK3CA amplification	50%	1.7	FFPE
P5	LUSC	M	66	IV	Treatment-naïve	FGFR3	c.746C>G(p.S249C)	36.85%	PIK3CA amplification	−	2.08	FFPE
P6	LUAC	M	74	IV	Treatment-naïve	FGFR3	c.746C>G(p.S249C)	0.86%	PIK3R2 c.1117G>A(p.G373R)	2.35%	−	Plasma
P7	LUSC	F	67	NA	Treatment-naïve	FGFR3	c.746C>G(p.S249C)	17.34%	PIK3CA c.1633G>A(p.E545K), c.2176G>A (p.E726K)	17.84%, 19.76%	−	FFPE
P8	LUSC	F	50	NA	Treatment-naïve	FGFR3	c.742C>T(p.R248C)	0.67%	−	−	−	FFPE
P9	LUSC	M	77	NA	Treatment-naïve	FGFR3	c.742C>T(p.R248C)	44.29%	−	−	−	FFPE
P10	LUAC	M	78	IV	Chemo, VEGFR antibody	FGFR3	c.742C>T(p.R248C)	0.62%	KRAS c.35G>A(p.G12D), HRAS c.38G>T (p.G13V), PIK3CA amplification	0.7%, 38.11%	1.9	FFPE
P11	LUAC	M	57	NA	Surgery	FGFR3	c.746C>G(p.S249C)	1.42%	KRAS c.35G>A(p.G12D), PIK3CA c.3103G>A(p.A1035T)	2.67%, 1.67%	−	FFPE
P12	LUSC	F	59	NA	Treatment-naïve	FGFR3	c.1138G>A(p.G380R)	8.52%	−	−	−	Plasma
P13	LUSC	M	55	NA	Treatment-naïve	FGFR3	c.746C>G(p.S249C)	15.18%	PIK3CA c.1633G>A(p.E545K)	18.82%	−	FFPE
P14	LUSC	M	61	NA	Treatment-naïve	FGFR3	c.1118A>G(p.Y373C)	87.37%	PIK3CA c.1633G>A(p.E545K)	46.84%	−	FFPE
P55	LUSC	M	65	NA	Chemo	FGFR2	c.1975A>G(p.K659E)	78.90%	PIK3CA amplification	−	3.63	FFPE
P56	LUSC	M	71	IV	Treatment-naïve	FGFR2	c.1977G>C(p.K659N)	2.86%	−	−	−	FFPE
P57	LUSC	M	74	NA	Treatment-naïve	FGFR2	c.1977G>C(p.K659N)	34.39%	PIK3CA c.3145G>C(p.G1049R)	21.98%	−	Plasma
P58	LUAC	F	64	NA	Chemo	FGFR2	c.868T>C(p.W290R)	17.12%	KRAS c.35G>T(p.G12V)	19.49%	−	Plasma
P59	LUAC	F	78	NA	Treatment-naïve	FGFR1	c.1638C>A(p.N546K)	3.15%	NRAS c.35G>A(p.G12D), NRAS c.182A>T (p.Q61L), PIK3CA c.2702G>T(p.C901F), c.323G>A(p.R108H), c.1357G>A(p.E453K), PTEN p.Y16X	0.385%, 1.22%, 1.7%, 0.54%, 2.06%, 4.05%	−	FFPE

LUAC, lung adenocarcinoma; LUSC, lung squamous cell carcinoma; TKI, tyrosine kinase inhibitor; PFS, progression-free survival; NA, not available; AF, allele frequency; CNV, copy number variation.

### The identification of novel *FGFR* fusions in NSCLC patients

**Figure 2:**
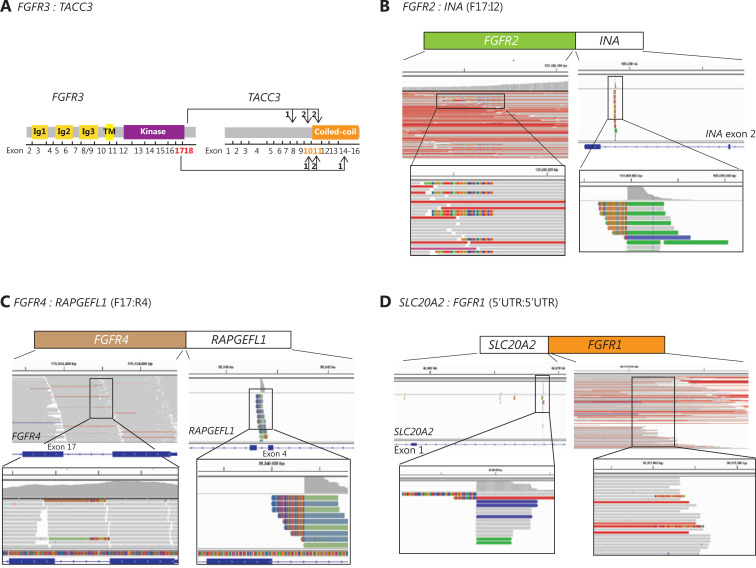
Visualization of *FGFR* fusions, including fusion partners, using the Integrative Genomics Viewer Browser. (A) The frequency of *FGFR3-TACC3* fusions in the cohort. (B-D) The IGV screenshots display the reads from next generation sequencing and reveal *FGFR* fusions of (B) *FGFR2-INA (F17:I2)*, (C) *FGFR4-RAPGEFL1 (F17:R4)*, and (D) *SLC20A1-FGFR1.*

*FGFR* fusions retaining the intact kinase domain were identified in 0.11% (12/10,966) of NSCLC patients examined (**Figure 1D** and **Table 2**). A majority of these patients (75%, 9/12) were positive for *FGFR3*-transforming acidic coiled-coil containing protein 3 gene (TACC3) fusions (*FGFR3-TACC3*), which were mostly reported in solid tumors^20^. Four of the 9 (45%) patients with *FGFR3-TACC3* fusions had 5′ breakpoints in *FGFR3* exon 17 and the remaining 55% were in exon 18, while *TACC3* exons 10 and 11 were the most common 3′ breakpoint locations (**Figure 2A**). We observed 1 case of *FGFR3* exon 17 fused to *TACC3* exon 14 that may have resulted in a fusion protein with compromised dimerization capacity due to a truncated coiled-coil domain (**Figure 2A**).

**Table 2:** The demographical and clinicopathological characteristics of patients who carried *FGFR* fusions encoding intact kinase domains

ID	Subtype	Gender	Age	Stage	Treatment history [TKI (PFS)]	Gene	Fusion	AF	Concurrent_alteration	AF_concurrent alt	CNV	Pre-treatment concurrent alt	Sample type
P15	LUAC	M	44	IV	Treatment-naïve	FGFR1	SLC20A2:5’UTR∼FGFR1:5’UTR	3.44%	−	−	−	−	Plasma
P16	LUAC	F	65	IV	Osimertinib (21 mo)	FGFR2	FGFR2:exon17∼INA:exon2	16.07%	EGFR p.746_750del, EGFR T790M, EGFR C797S	20.6%, 6.35%, 1.53%	−	EGFR p.746_750del, EGFR T790M	Plasma (post osimertinib)
P17	LUSC	M	54	IV	Chemo, icotinib (7 mo), osimertinib (5 mo)	FGFR3	FGFR3:exon18∼TACC3:exon10	1.70%	EGFR p.746_750del, EGFR T790M, EGFR amplification	4.8%, 0.2%	1.82	EGFR p.746_750del	Plasma (post osimertinib)
P18	LUSC	M	57	II/III	Treatment-naïve	FGFR3	FGFR3:exon18∼TACC3:exon10	26.72%	−	−	−	−	FFPE
P19	LUAC	F	40	IV	Treatment-naïve	FGFR3	FGFR3:exon17∼TACC3:exon10	2.28%	−	−	−	−	Plasma
P20	LUSC	M	68	IV	Treatment-naïve	FGFR3	FGFR3:exon17∼TACC3:exon11	23.81%	EGFR T790M	0.43%	−	−	FFPE
P21	LUAC	F	34	III	Gefitinib (7 mo), osimertinib (10 mo)	FGFR3	FGFR3:exon17∼TACC3:exon14	1.17%	EGFR p.E746_A750del	6.52%	−	−	Plasma (post osimertinib)
P22	LUAC	M	44	IV	Chemo, erlotinib (10 mo), osimertinib (10 mo), immunotherapy	FGFR3	FGFR3:exon17∼TACC3:exon11	30.30%	EGFR p.L747_P753delinsS, EGFR T790M, PIK3CA H1047R, EGFR amplification	80.5%, 2.83%, 29.37%	3.3	−	Plasma (post erlotinib)
P23	LUAC	M	38	IV	Treatment-naïve	FGFR3	FGFR3:exon18∼TACC3:exon11	2.74%	−	−	−	−	Plasma&Tissue
P24	LUSC	F	58	III	Surgery	FGFR3	FGFR3:exon18∼TACC3:exon8	7.12%	−	−	−	−	FFPE
P25	LUSC	M	68	NA	Treatment-naïve	FGFR3	FGFR3:exon18∼TACC3:exon11	1.38%	−	−	−	−	Plasma
P26	LUAC	F	48	IV	Treatment-naïve	FGFR4	FGFR4:exon17∼RAPGEFL1:exon4	4.04%	EGFR p.L747_E749del	21.71%	−	−	FFPE

LUAC, lung adenocarcinoma; LUSC, lung squamous cell carcinoma; TKI, tyrosine kinase inhibitor; PFS, progression-free survival; NA, not available; AF, allele frequency; CNV, copy number variation.

We also observed 1 gene rearrangement event involving *FGFR2* and an internexin neuronal intermediate filament protein α gene (*INA*) fusion (*FGFR2 F17: INA I2*) in a patient (P16) with stage IV lung adenocarcinoma (**Figure 2B**). The *FGFR2-INA* fusion was previously reported in low grade gliomas that drove oncogenesis *via* MAPK and PI3K/mTOR pathway activation^21^. Our observations represented the first case of a *FGFR2-INA* fusion in NSCLC, in particular, lung adenocarcinoma. Furthermore, 1 gene fusion event involving fibroblast growth factor receptor 4 (*FGFR4*) and the Rap guanine nucleotide exchange factor like 1 gene (*RAPGEFL1*) (*FGFR4 F17: RAPGEFL1 R4*) was detected in a lung adenocarcinoma patient (P26) (**Figure 2C**), which has not been previously documented, and therefore further validation of its function is necessary in future research. Notably, a concurrent activating* EGFR* ex19del mutation was also detected at an allele frequency of 21.71% in this patient. In addition, we observed 1 patient with a 5′-untranslated region of the Solute Carrier Family 20 Member 2 gene (*SLC20A2*) fused to *FGFR1* exon 17 (**Figure 2D**).

Of note, concomitant *EGFR* mutations or *EGFR* amplifications were observed in 6 of the 12 *FGFR* fusion patients (**Table 2**), 4 of which were previously treated with EGFR TKIs, but the disease had progressed prior to NGS tests. Although half the patients (*n* = 2) did not have pretreatment samples, the remaining 2 patients (P16 and P17) likely acquired *FGFR* fusions as alternative mechanisms to combat the anti-tumor activity of EGFR TKIs (**Table 2**). Furthermore, a concurrent *PIK3CA* H1047R mutation was observed in 1 patient (P22) and may also have acted as a mechanism of acquired resistance to prior therapies including TKIs (**Table 2**). No other known dominant driver mutations were detected in the remaining 6 patients (**Table 2**).

### Amplification of the *FGF1*9 and *FGFR* genes in NSCLC patients

As previously mentioned, we observed more amplification events in *FGFR1* than other *FGFRs* (**Figure 1B**). *FGFR* amplification was detected in a total of 24 patients, a majority of which (87.5%, 21/24) were *FGFR1* amplifications (**Figure 1D**). Similarly, the majority of *FGFR*-amplified patients (67%) were LUSC and 92% were male (**Table 3**). Notably, 25 patients (12%, 25/210) had multiple alterations in *FGFR* genes, but oncogenic *FGFR* mutations, fusions, or gene amplifications were almost mutually exclusive events, with the exception that 4 *FGFR3*-mutant patients had concurrent *FGFR1* amplifications (**Figure 1D**). Two patients had concurrent *EGFR* activating mutations and received prior EGFR-TKI treatments. However, no pretreatment samples were available for mutation profiling for these patients. The remaining patients (92%, 22/24) had no other dominant driver mutations and were either chemotherapy-refractory or treatment naïve (**Table 3**).

**Table 3:** The demographical and clinicopathological characteristics of patients who had *FGFR* and *FGF19* amplifications

ID	Subtype	Gender	Age	Stage	Treatment history [TKI (PFS)]	Gene	CNV	Concurrent alteration	AF_concurr-ent alt	Sample type
P27	LUAC	F	48	IV	Chemo, icotinib (quick PD), osimertinib	FGFR4	1.72	−		Plasma (post osimertinib)
P1	LUSC	M	56	NA	Surgery, chemo	FGFR1	1.88	−		Plasma
P28	LUAC	M	73	NA	Surgery, gefitinib (17 m), osimertinib (quick PD), afatinib (5 m)	FGFR2	2.3	EGFR p.E746_S752delinsA, EGFR p. G724S, PIK3CA p.E545K	16.22%, 17.27%, 18.44%	FFPE (post afatinib)
P29	LUAC	M	62	IV	Treatment-naïve	FGFR1	1.78	−		FFPE
P30	LUSC	M	70	NA	Treatment-naïve	FGFR1	6.56	−		FFPE
P31	LUAC	M	62	NA	Treatment-naïve	FGFR1	1.71	−		FFPE
P32	LUAC	M	60	IV	Treatment-naïve	FGFR1	5.14	−		FFPE
P33	LUSC	M	52	NA	Chemo, radiotherapy, anlotinib (PR)	FGFR1	2.19	−		Plasma
P34	LUSC	M	52	NA	Chemo, radiotherapy, anlotinib (PR)	FGFR1	2.75	−		FFPE
P35	LUSC	M	60	NA	Chemo, nivolumab (quick PD)	FGFR3	2.08	−		FFPE
P5	LUSC	M	66	IV	Treatment-naïve	FGFR1	3.48	−		FFPE
P36	LUSC	M	53	NA	Surgery, chemo	FGFR1	2.45	−		FFPE
P37	LUSC	M	65	III	Treatment-naïve	FGFR1	2.55	−		Tissue
P38	LUAC	M	69	NA	NA	FGFR1	2.73	−		FFPE
P39	LUSC	M	73	NA	Chemo	FGFR1	4.15	−		FFPE
P9	LUSC	M	77	NA	Treatment-naïve	FGFR1	7.24	−		FFPE
P10	LUAC	M	78	IV	Chemo, VEGFR mAb	FGFR1	1.99	−		FFPE
P40	LUAC	F	48	IV	Chemo, gefitinib (5 m, PD), osimertinib (10 m, PD)	FGFR1; FGFR4	2.33; 2.51	EGFR p.E746_A750del, p. T790M, p. C797S	72.43%, 3.91%, 29.68%	Pleural effusion (post osimertinib)
P41	LUSC	M	70	NA	Chemo	FGFR1	3.51	−		FFPE
P42	LUAC/SC	M	72	NA	Treatment-naïve	FGFR1	1.93	−		FFPE
P43	LUSC	M	68	NA	Treatment-naïve	FGFR1	2.33	−		FFPE
P44	LUSC	M	55	NA	Treatment-naïve	FGFR1	2.06	PTEN p.L316NfsX4	60.41%	Tissue
P45	LUSC	M	68	NA	Treatment-naïve	FGFR1	2.09	PIK3CA p.D843Y, p.F1039L, p.M1043I, EGFR p.G796C	1.29%, 1.33%, 0.82%, 0.88%	FFPE
P46	LUSC	M	52	NA	Treatment-naïve	FGFR1	3.17	−		FFPE
P47	LUSC	M	62	II	Chemo	FGF19	1.77	−		FFPE
P48	LUAC	M	62	IV	Treatment-naïve	FGF19	1.91	−		FFPE
P49	LUSC	M	61	IV	Chemo	FGF19	3.02	−		Plasma
P50	LUSC	M	65	NA	Chemo	FGF19	8.34	PIK3CA amplification	3.63%	FFPE
P51	LUAC	F	84	NA	Treatment-naïve	FGF19	2.91	−		Pleural effusion
P52	LUAC	M	60	IV	Treatment-naïve	FGF19	12.88	−		FFPE
P6	LUAC	M	74	IV	Treatment-naïve	FGF19	6.99	PIK3R2, c.1117G>A(p.G373R)	2.35%	Plasma
P53	LUAC	M	73	NA	Treatment-naïve	FGF19	9.76	−		FFPE
P54	LUSC	M	68	NA	Treatment-naïve	FGF19	3.09	−		FFPE

LUAC, lung adenocarcinoma; LUSC, lung squamous cell carcinoma; TKI, tyrosine kinase inhibitor; PFS, progression-free survival; NA, not available; AF, allele frequency; CNV, copy number variation.

We also identified 9 patients (0.08%, 9/10,966) who had amplifications of *FGF19* (**Figure 1D**), which encodes a unique, high affinity ligand that specifically binds to FGFR4 in a heparin-dependent manner. Our observations were consistent with previous studies reporting on the role of the FGF19-FGFR4 signaling axis in human cancers, including hepatocellular carcinoma^22^ and lung squamous cell carcinoma^23^. Two patients had concomitant aberrations of the PI3K signaling pathway, including *PIK3CA* amplification and the *PIK3R2* G373R missense mutation (**Table 3**). All patients were either chemotherapy-refractory or treatment naïve.

## Discussion

This study represented the first comprehensive survey of *FGFR* aberrations in a large population of Chinese patients with NSCLC. Approximately 1.9% of the population had *FGFR* aberrations, including point mutations, gene rearrangements, and amplifications, with the most common abnormality being FGFR point mutations. The prevalence of FGFR alterations in this Chinese NSCLC population was relatively lower than that of a prior study (5.7%), as reported by Helsten et al.^24^ in which the study population was unlikely to be only Chinese. Currently, there are a number of FGFR inhibitors approved by the Federal Drug Administration (FDA), including ponatinib, regorafenib, pazopanib, lenvatinib, and nintedanib, which were included in a trial specifically targeting NSCLC patients^25^. All these FGFR inhibitors are multi-kinase inhibitors that also exhibit nonspecific anti-tumor activities against other tyrosine kinases, including VEGFR, PDGFR, ROS1, and/or RET. However, there are also specific FGFR inhibitors in clinical development. Notably, erdafitinib, a functionally selective pan-FGFR inhibitor, has been approved by the FDA to treat advanced metastatic urothelial cancers^6,8^. Different *FGFR* abnormalities responded differently to erdafitinib, with the highest response rate seen for patients with FGFR point mutations^8^. Another selective FGFR inhibitor, pemigatinib, was also recently granted accelerated approval for treatment of late stage *FGFR2+* cholangiocarcinoma patients^26^. It is definitely of great clinical interest to study these FGFR inhibitors in NSCLC patients, so future trials may be warranted.

Unlike lung adenocarcinomas, no targeted molecular therapies have been developed for squamous cell lung cancers because targetable oncogenic aberrations are scarce in this tumor type. Here, we report that *FGFR* aberrations were present in approximately 6.8% of the LUSC cohort of this study, which was higher than the frequency (1.3%) in LUAC patients. Notably, over 75% of *FGFR1* amplification events were observed in LUSC patients, which is consistent with previous findings^24,27^. More than half of the patients who carried *FGFR* activating/transforming mutations had concurrent dominant mutations in PI3K pathway genes, including *PIK3CA* and *PIK3R2*, consistent with previous reports^28–30^. Furthermore, we reported the overlapping of activated *FGFR* genes and genetic alterations of the PI3K pathway in NSCLC, including both LUAC and LUSC. A prior study by Packer et al.^31^ revealed that PI3K inhibitors enhanced the anti-tumor efficacies of anti-FGFR inhibitors *in vitro* in endometrial cancers in which the activation of the PI3K pathway was observed in > 90% of *FGFR2*-mutated cases. The activation of the PI3K pathway was also reported to be enriched in breast cancer patients with activated *FGFR/FGF* signaling^32^. Together, our findings highlighted an intriguing molecular feature and potential therapeutic target for combination therapies targeting the FGFR and PI3K pathways in *FGFR*-positive NSCLC patients exhibiting activated PI3K and MAPK pathways.

Furthermore, we identified a total of 12 *FGFR* gene rearrangements in the NSCLC population that maintained intact FGFR kinase domains. *FGFR* fusions did not segregate well by histology or sex, as was previously reported by Wang et al.^33^ which was likely due to the restricted cohort size. The majority of these patients were *FGFR3-TACC3* positive, but we also observed 1 case of a *FGFR2-INA* fusion that was originally described in gliomas, and 2 novel *FGFR* fusions, including *SLC20A2-FGFR1* and *FGFR4-GAPGEFL1*. A prior study by Wu et al.^34^ reported a case of prostate cancer with the *SLC45A3* non-coding exon 1 fused to the intact coding region of *FGFR2,* in which the *SLC45A3-FGFR2* fusion was predicted to drive the overexpression of wildtype *FGFR2*. Thus, the *SLC20A2-FGFR1* fusion observed in the current study may also have been able to drive the overexpression of wildtype *FGFR1*, although additional studies are needed to test this possibility. It is worth noting that half (*n* = 6) of the *FGFR* fusion patients carried *EGFR* aberrations, including *EGFR* ex19del, T790M, C797S, and *EGFR* amplifications. Two-thirds of those patients received prior EGFR TKI therapies. Reminiscent of a prior report by Ou et al.^35^, this observation suggested that* FGFR* fusions may act as a mechanism of acquired resistance to EGFR inhibitors in patients (P16, P17, P21, and P22) who were previously treated with EGFR TKIs.

Aside from point mutations and gene rearrangements, approximately 15% of all *FGFR* aberrations were amplifications, with *FGFR1* amplifications being the most common anomalies. *FGFR* amplifications predominated in LUSC patients at a prevalence of 1.6%, in contrast to that of < 0.1% in the LUAC population. These frequencies were relatively lower than those reported by Helsten et al.^24^ (9% and 4%, respectively), which could be attributed to a number of reasons including the ethnic differences underlying these two study populations, the restricted NSCLC cohort size of Helsten et al., as well as the inclusion of cases who had only liquid biopsy ctDNA samples in this work.

Previous studies have shown that *FGFR1* amplification was common in breast cancer patients with early relapses and poor clinical outcomes^36^. Therefore, antibodies targeting FGFR represent a valid therapeutic strategy to treat breast cancer or other cancer histologies, including NSCLC. In addition, we also observed a low frequency of *FGF19* amplifications in our NSCLC population. FGF19 encodes the ligand for FGFR4, and it was previously shown that *FGF19* amplifications corresponded with constitutive activation of FGF receptor 4 (FGFR4)-dependent ERK/AKT-p70S6K-S6 signaling activation in head and neck squamous carcinoma cells^37^; thus, raising the question as to whether the FGF19/FGFR4 axis also acts as an oncogenic driver in these NSCLC patients and represents a therapeutic target.

## Conclusions

This study reported the frequency of *FGFR* aberrations, including activating mutations, gene rearrangements, and gene amplifications in a large population of Chinese NSCLC patients, and revealed the potential clinical utility of targeting *FGFR* aberrations with FGFR inhibitors in NSCLC patients. We also reported novel *FGFR* fusion events in NSCLC patients, including *SLC20A2-FGFR1*, *FGFR2-INA*, and *FGFR4-GAPGEFL1*; thus, highlighting potential therapeutic targets for the management of such patients.
