# Supplementary material for: Targeting FGFR in non-small cell lung cancer: implications from the landscape of clinically actionable aberrations of FGFR kinases
Source: Cancer Biol Med. 2021 Jun 15;18(2):490–501. doi: 10.20892/j.issn.2095-3941.2020.0120 (PMC8185861; doi:10.20892/j.issn.2095-3941.2020.0120)
Supplement: Supplementary file 1 [file cbm-18-490-s001.pdf]

Supplementary materials

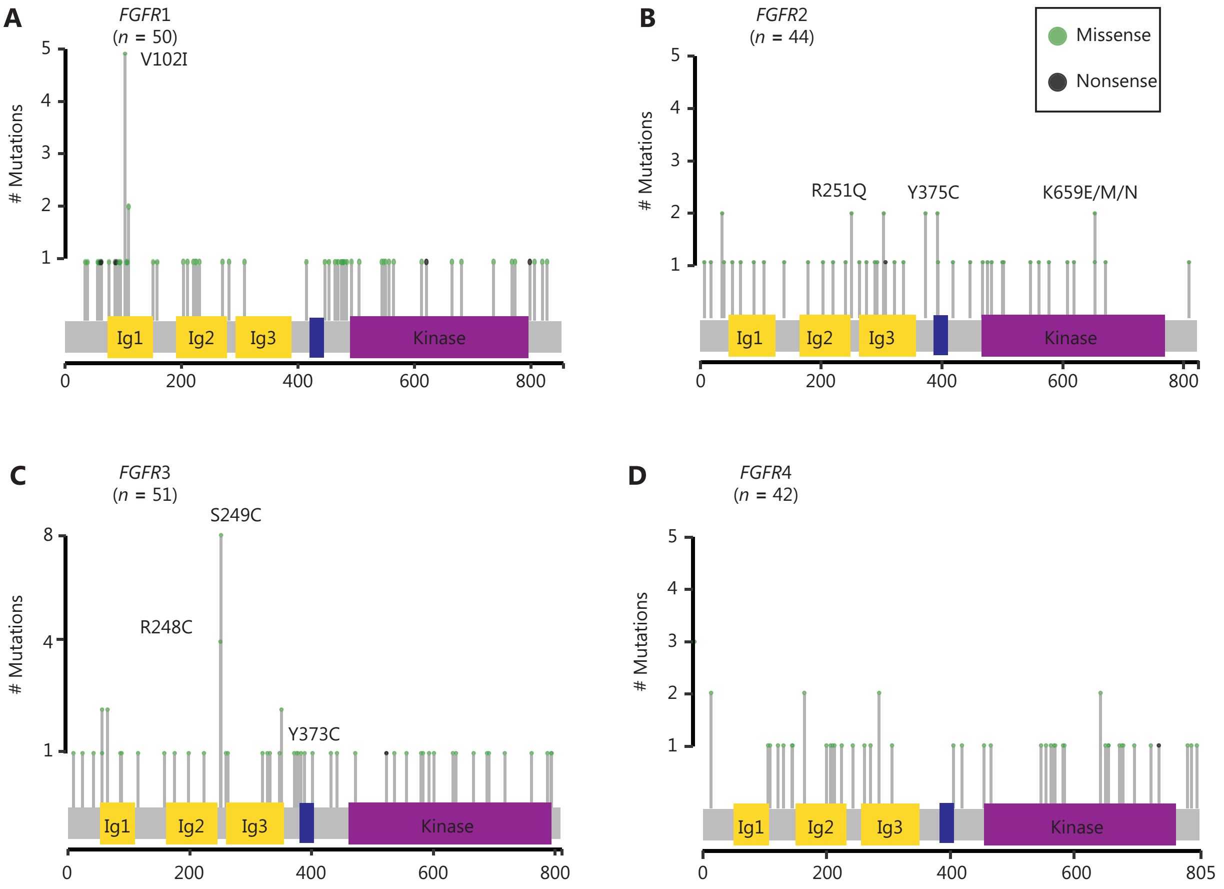

Figure S1 Distribution of all *FGFR* mutations identified in the Chinese NSCLC population.

Table S1 List of *FGFR1-4* oncogenic and likely oncogenic mutations according to OncoKB annotations

FGFR1	FGFR2	FGFR3	FGFR4
K687E	A648T	A391E	D127H
N577K	C382R	D641G	N535K
V592M	D101Y	D641N	V550E
	D530N	G370C	Y367C
	E219K	G375C	
	E565G	G380R	
	E731K	G382D	
	F276C	I538V	
	G271E	K650E	
	I547V	K650M	
	I642V	K650N	
	K525E	K650Q	
	K526E	K650R	
	K641N	K650T	
	K641R	M528I	
	K659E	N540K	
	K659N	N540S	
	L617F	R248C	
	L617M	R669G	
	M391R	S249C	
	M535I	S371C	
	M537I	V555M	
	N549H	Y373C	
	N549K	Y647C	
	N549S		
	N549T		
	P253R		
	R203C		
	R251Q		
	R678G		
	S252W		
	S267_D273dup		
	T341P		
	V564F		
	V564I		
	W290R		
	W290_I291delinsC		
	Y375C		
	Y381D		

Continued
